# Examination of sub-harmonic responses along with various initial conditions induced by multi-staged clutch damper system

**DOI:** 10.1038/s41598-022-15470-6

**Published:** 2022-07-05

**Authors:** Jong-Yun Yoon, Byeongil Kim

**Affiliations:** 1 Department of Mechatronics Engineering, Incheon National University, Incheon, 22012 Republic of Korea; 2 School of Mechanical Engineering, Yeungnam University, Gyeongsan, 38541 Republic of Korea

**Keywords:** Engineering, Mechanical engineering

## Abstract

Using the harmonic balance method to investigate the nonlinear dynamic behaviors pertaining to sub-harmonic responses is difficult compared with that of super-harmonic cases because of the limitations of the HBM. Since sub-harmonic motions differ under various initial conditions, difficulties can arise when this method is used to calculate all possible solutions within sub-harmonic resonances. To explore complex dynamic behaviors in sub-harmonic resonant areas, this study suggests mathematical and numerical techniques to estimate sub-harmonic responses depending on various initial conditions. First, sub-harmonic responses are calculated under various excitation conditions relevant to the sub-harmonic input locations of the HBM formula. Second, the HBM results are verified by comparing them with the results of the numerical simulation (NS) under various initial conditions with respect to different frequency up-sweeping paths. Finally, the positive real part of the eigenvalues is examined to anticipate bifurcation characteristics, which reflect the relevance of the complex dynamic behaviors in the eigenvalues’ unstable solutions. Overall, this study successfully proves that the techniques and methods described are suitable for examining complex sub-harmonic responses, and suggests basic ideas for analyzing nonlinear dynamic behaviors in sub-harmonic resonances using the HBM.

## Introduction

**Figure 1:**
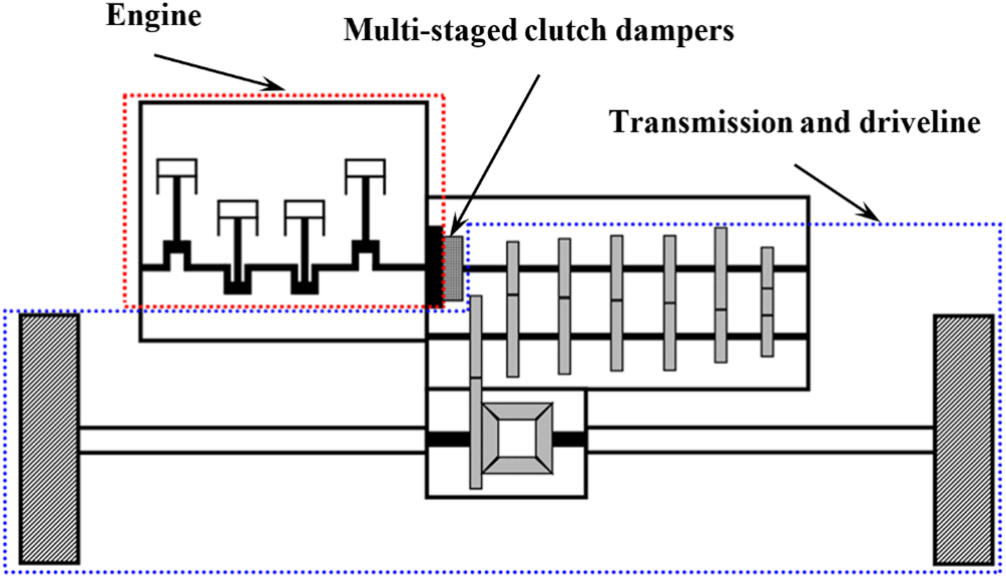
A physical driveline system based on the front-engine and front-wheel layout with multi-staged clutch dampers^22^.

Nonlinear dynamic responses observed in a practical system, as illustrated in Fig. 1, reflect various types of dynamic phenomena, generally called super- and sub-harmonic, periodic, quasi-periodic, and chaotic. These responses are normally signified by various types of bifurcations in super- and sub-harmonic resonant areas. Complex nonlinear dynamic behaviors in sub-harmonic resonances are particularly difficult to find, relative to other resonances such as primary and super-harmonic areas, when the harmonic balance method (HBM) is employed.

The methods for solving complex nonlinear problems using the HBM have been reported for decades^1–21^. For example, Peng et al. implemented nonlinear output frequency response functions (NOFRFs) in strong nonlinear equations by applying the Volterra series to extend the classic frequency response function (FRF) to a nonlinear case^1^. Al-shyyab and Kahraman investigated sub-harmonic and chaotic motions in a multi-mesh gear train using a nonlinear time-varying dynamic model^2^. Here, nonlinear dynamic motions were simulated with the multi-term harmonic balance method and correlated with the direct numerical integration results. Chen et al. used the incremental harmonic balance (IHB) method to investigate the limit cycle oscillation of a two-dimensional airfoil with parameter variability in an incompressible flow^3^, and further utilized the IHB to estimate the strong nonlinear cubic stiffness, subject to either (1) non-probability but bounded uncertainty, or (2) bounded stochastic parameters. Kim et al. proposed and verified a multi-term harmonic balance method by including adaptive arc-length continuation and stability calculation capabilities^4^, which aided them in developing nonlinear frequency response calculations of a torsional system with clearance-type nonlinearity. Miguel et al. suggested the closed-form solutions for the Bouc-Wen and LuGre models by developing a smoothing procedure with the harmonic balance method^5^. The bifurcation analysis techniques with the employment of the harmonic balance method were suggested by Detroux et al. and Xie et al.^6,7^. The basic mathematical models of the harmonic balance method by using Galerkin forms were investigated by the prior researches^8–11^. Masiani et al. suggested masing model to analyze the hysteretic behavior of the elements with the multi-component harmonic balance method^12^. Raghothama and Narayanan investigated the bifurcation and chaos which occur in geared rotor bearing and piecewise nonlinear stiffness systems by using the incremental harmonic balance method^13,14^. Shen et al. established the dynamic model of a spur gear pair by including the backlash, time-varying stiffness and static transmission error^15^. Wong et al. presented the nonlinearities in the restoring force under the unsymmetrical piecewise-linear stiffness^16^. To determine the stability of system responses, the Hill’s method was suggested and employed^17,18^. Comparin and Singh suggested the driveline model embedded by the clearance type nonlinearities and investigated its complex system responses^19^. Sundararajan and Noah examined the nonlinear responses in rotor systems by employing shooting/arc-length continuation method^20,21^.

**Figure 2:**
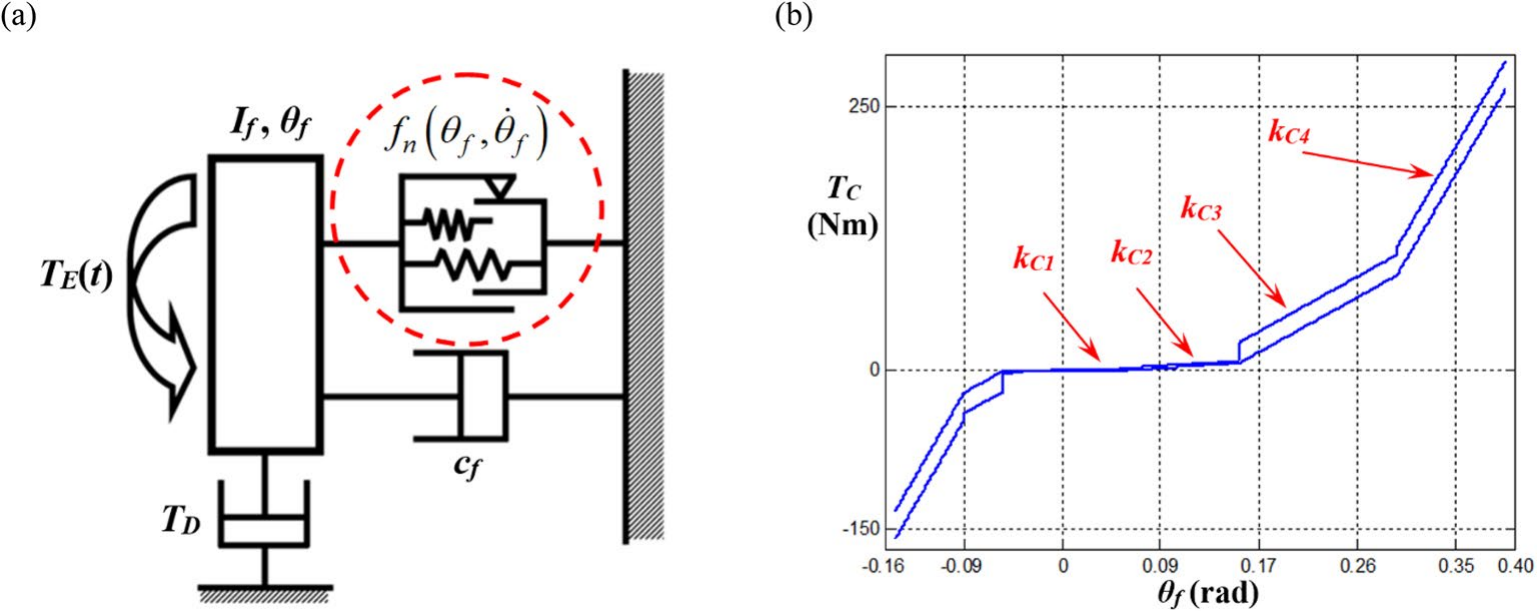
A single-degree-of-freedom system with piecewise-type nonlinearities based on a physical system: (**a**) a nonlinear torsional system model with one DOF; (**b**) Torque *T*_*C*_(*δ*_1_) profile for a multi-staged clutch damper^8^.

However, some difficulties remain in capturing the complex motions of sub-harmonic responses, especially when their dynamic behaviors differ depending on various initial conditions. Thus, to investigate sub-harmonic responses, the sub-harmonic index must be used, and basic matrix formulae are constructed^8,18^. To capture sub-harmonic responses, further techniques must be utilized, such as numerical modification of the input conditions^18^. This study expands upon the theoretical and numerical methodology of prior studies which could not clearly determined the sub-harmonic responses, by investigating the nonlinear dynamic behaviors in sub-harmonic resonant areas sensitive to initial conditions using the HBM^8^. The specific objectives are as follows: first, to investigate nonlinear dynamic motions under various input excitation conditions when the sub-harmonic input components of the HBM are artificially defined; second, to verify sub-harmonic responses of the HBM by comparing the numerical simulation (NS) under various initial conditions and different frequency up-sweeping paths; and finally, to examine the eigenvalues’ unstable solutions for bifurcation characteristics, which will provide a guide for anticipating bifurcation characteristics while the stability conditions are determined simultaneously based on the Hill’s method. Therefore, understanding the dynamic behaviors at sub-harmonic resonance areas will give the reasonable insights to resolve the severe vibrational problems such as gear rattle under the vehicle coast conditions^8,22^. To analyze these main issues, this study focuses on a torsional system with one degree-of-freedom (DOF) affected by piecewise-type nonlinearities, as presented in Fig. 2. This system has been simplified from the physical system shown in Fig. 1.

## Physical system and its problem formulation

### Physical system and its modeling

Figure 1 illustrates a physical driveline system based on the front-engine and front-wheel (FF) layout^22^. The entire system can be considered to have three main parts: the engine, which is the power generating system; the multi-staged clutch dampers, which operate as the vibration isolator; and the transmission and driveline subsystems, which transfer the torque from the engine into the wheel. Assuming the inertia value of the transmission and driveline systems’ lumped mass is relatively higher than the engine inertia, the physical system can be simplified to a single DOF system, as illustrated in Fig. 2a^8,22^. The multi-staged clutch dampers have the properties listed in Table 1^8,22^, and are the main nonlinearities for the system, whereas the transmission and driveline systems are assumed as the ground. Using the values provided in Table 1 which were measured from the clutch damper experimental setup, the clutch torque profile is drawn, as shown in Fig. 2b. Therefore, based on prior studies, the nonlinear one DOF torsional system can be considered a part of the driveline by focusing on the rotational motion observed in the crankshaft and flywheel’s lumped mass with multi-staged clutch dampers^8,22,23^. In this study, the inertia of the flywheel and crankshaft’s lumped mass, *I*_*f*_  = 1.38 × 10^−1^ kg m^2^, and viscous damping, *c*_*f*_ = 1.59 N m s/rad^8^ were the parameters implemented for the torsional system shown in Fig. 2a, and the input torque, *T*_*E*_, drag torque, *T*_*D*_, and clutch torque, $f_{n} \left( {\theta_{f} ,\dot{\theta }_{f} } \right)$ define the additional parameters. Here, $\theta_{f}$ and $\dot{\theta }_{f}$ are the angular displacement and velocity of the flywheel (subscript *f*), respectively, as indicated in Fig. 2a. Here, the employed system parameters are measured and given based on the manual transmission type of driveline system^23^.

**Table 1:** Properties for the piecewise type nonlinearities based on the practical system^8,23^.

Property	Stage	Value
Torsional stiffness, *k*_*Ci*_ (Linearized in a piecewise manner) (Nm/rad)	1	10.1
	2	61.8
	3	595.8
	4	1838.0
Hysteresis, *H*_*i*_ (Nm)	1	0.98
	2	1.96
	3	19.6
	4	26.5
Transition angle at positive side (*θ*_*f*_ > *0*), $\phi_{pi}$ (rad)	1	0.05
	2	0.16
	3	0.30
	4	0.39
Transition angle at negative side (*θ*_*f*_ < *0*), $\phi_{ni}$ (rad)	1	− 0.04
	2	− 0.05
	3	− 0.09
	4	− 0.15

### Development of a mathematical model with piecewise-type nonlinearities

The equation of motion for the one DOF system shown in Fig. 2a is derived as follows:

1 $$I_{f} \ddot{\theta }_{f} \left( t \right) + c_{f} \dot{\theta }_{f} \left( t \right) + f_{n} \left( {\theta_{f} ,\dot{\theta }_{f} } \right) = T_{E} \left( t \right) - T_{D}$$

Here, $f_{n} \left( {\theta_{f} ,\dot{\theta }_{f} } \right)$ is the nonlinear function, which will be explained in terms of piecewise-type nonlinearities. *T*_*E*_(*t*) and *T*_*D*_ are the sinusoidal input and drag torque, respectively. The excitation of the system in terms of its input torque is given by Fourier coefficients from the measured data as follows:

2 $$T_{E} \left( t \right) = T_{m} + \mathop \sum \limits_{i = 1}^{{N_{\max } }} T_{pi} cos\left( {i\omega_{p} t + \varphi_{pi} } \right)$$

Here, *T*_*m*_ and *T*_*pi*_ are the mean and alternating parts of the input torque, respectively; $\omega_{p}$ and $\varphi_{pi}$ are the excitation frequency and phase angle, respectively; and *N*_*max*_ is the maximum number of harmonics correlated to the harmonic index of the HBM. The input torques are described using the properties listed in Table 2 and, assuming the system is under steady-state conditions, the drag torque can be expressed as *T*_*D*_ = *T*_*m*_, also the employed number of alternating part of toque is 1 with *T*_*pi*_ (*i* = 1). Here, the torque profile employed as shown in Table 2 is measured from the engine dynamometer setup under the WTO (wide open throttle) conditions based on the 4-cylinder engine^23^.

**Table 2:** Employed input torque profiles from the engine dynamometer test.

Torque Component		
Magnitude (Nm)		Phase (rad)
*T*_*m*_	*T*_*p1*_	
168.9	251.5	− 1.93

In addition, the clutch torque is dependent on various factors, while the input torque is transferred from the engine into the rest of the driveline system^8^. The basic profile of piecewise-type nonlinearities induced by multi-staged clutch dampers is shown in Fig. 2b, where *k*_*Ci*_ (*i* = 1,2,3,4) indicates the stage of stiffness. The main factors to consider for the clutch torque are the clutch torque *T*_*S*_ from multi-staged linear springs, a second clutch torque *T*_*H*_ from the dry friction between the clutch plate and friction materials, and the total preload effect *T*_*SPr*_ due to design concepts under various practical conditions. The mathematical model of nonlinear torque $f_{n} \left( {\theta_{f} ,\dot{\theta }_{f} } \right)$ (or *T*_*C*_) can be derived from prior studies^8,22,23^. First, the clutch torque $T_{S} \left( {\theta_{f} } \right)$ from the stiffness with a smoothing factor $\sigma_{C}$ of $1 \times 10^{3}$ is described as follows:

3a $$T_{S} \left( {\theta_{f} } \right) = k_{C1} \theta_{f} + \frac{1}{2}\mathop \sum \limits_{i = 2}^{N} \left( {k_{C\left( i \right)} - k_{{C\left( {i - 1} \right)}} } \right)\left( {T_{{sp\left( {i - 1} \right)}} - T_{{sn\left( {i - 1} \right)}} } \right),$$

3b $$T_{sp\left( i \right)} = \left( {\theta_{f} - \phi_{p\left( i \right)} } \right)\left[ {tanh\left\{ {\sigma_{C} \left( {\theta_{f} - \phi_{p\left( i \right)} } \right)} \right\} + 1} \right],$$

3c $$T_{sn\left( i \right)} = \left( {\theta_{f} + \phi_{n\left( i \right)} } \right)\left[ {tanh\left\{ {\sigma_{C} \left( {\theta_{f} + \phi_{n\left( i \right)} } \right)} \right\} - 1} \right],$$

where *k*_*C*(*N*)_ (or *k*_*C*(*i*)_) is the *N*th (or *i*th) stage of the clutch stiffness (with subscript *N* or *i*), *T*_*sp*(*i*)_ (or *T*_*sn*(*i*)_) is the positive (or negative) side of the clutch torque induced by the stiffness at the *i*th stage (with subscript *p* or *n*), and $\phi_{p\left( i \right)}$ (or − $\phi_{n\left( i \right)}$) is the *i*th transition angle of the positive (or negative) side. Second, the clutch torque *T*_*H*_ induced by dry friction is derived with a smoothing factor $\sigma_{H}$ of 0.1.

4a $$T_{H} \left( {\theta_{f} ,\dot{\theta }_{f} } \right) = \frac{{H_{\left( N \right)} }}{2}tanh\left( {\sigma_{H} \dot{\theta }_{f} } \right) + \mathop \sum \limits_{i = 2}^{N} \left( {\frac{{H_{\left( i \right)} }}{4} - \frac{{H_{{\left( {i - 1} \right)}} }}{4}} \right)\left[ {T_{{Hp\left( {i - 1} \right)}} + T_{{Hn\left( {i - 1} \right)}} } \right],$$

4b $$T_{Hp\left( i \right)} = tanh\left\{ {\sigma_{C} \left( {\theta_{f} - \phi_{p\left( i \right)} } \right)} \right\}\left[ {1 + tanh\left( {\sigma_{H} \dot{\theta }_{f} } \right)} \right],$$

4c $$T_{Hn\left( i \right)} = tanh\left\{ {\sigma_{C} \left( {\theta_{f} + \phi_{n\left( i \right)} } \right)} \right\}\left[ {1 - tanh\left( {\sigma_{H} \dot{\theta }_{f} } \right)} \right],$$

where *H*_*N*_ (or *H*_(*i*)_) is the *N*th (or *i*th) stage of hysteresis (with subscript *N* or *i*), and *T*_*Hp*(*i*)_ (or *T*_*Hn*(*i*)_) is the positive (or negative) side of the clutch torque induced by hysteresis at the *i*th stage (with subscript *p* or *n*). In addition to calculating the torque using Eqs. (3) and (4), the preload *T*_*Pri*_ (subscript *i* = 1(or 2) for the positive (or negative) value) must be considered as a function of $\theta_{1pr}$.

5a,b $$T_{SPr} \left( {\theta_{1pr} } \right) = \frac{1}{2}T_{Pr1} \left[ {tanh\left( {\sigma_{C} \theta_{1pr} } \right) + 1} \right] + \frac{1}{2}T_{Pr2} \left[ { - tanh\left( {\sigma_{C} \theta_{1pr} } \right) + 1} \right],{ }\theta_{1pr} = \theta_{f} - \phi_{Pr} .$$

Here, *T*_*SPr*_ is the total clutch torque induced by the preload, *T*_*Pr1*_ (or *T*_*Pr2*_) is the positive (or negative) torque induced by the preload, and $\phi_{Pr}$ is the angle located at the preload. Thus, the total clutch torque is estimated by the summation of $T_{S} \left( {\theta_{f} } \right)$, $T_{H} \left( {\theta_{f} ,\dot{\theta }_{f} } \right)$, and $T_{SPr} \left( {\theta_{1pr} } \right)$ from Eqs. (3)–(5), as follows:

6 $$f_{n} \left( {\theta_{f} ,\dot{\theta }_{f} } \right) = T_{C} \left( {\theta_{1pr} ,\dot{\theta }_{1pr} } \right) = T_{S} \left( {\theta_{1pr} } \right) + T_{H} \left( {\theta_{1pr} ,\dot{\theta }_{1pr} } \right) + T_{SPr} \left( {\theta_{1pr} } \right).$$

Figure 2b shows the physical clutch torque profile well obtained using Eqs. (3)–(6).

### Development of the HBM model based on the Galerkin scheme

The Galerkin scheme in Eq. (1) can be expressed as follows^8,22^:

7 $$- \omega^{2} m\underline{\underline{{{\text{HP}}}}}^{\prime \prime } \underline{{\theta_{{\text{c}}} }} + \omega c\underline{\underline{{{\text{HP}}}}}^{\prime } \underline{{\theta_{{\text{c}}} }} + \underline{{{\text{f}}_{{\text{n}}} }} \left( {\underline{{\theta_{{\text{f}}} }} ,\underline{{\dot{\theta }_{{\text{f}}} }} } \right) - \underline{{{\text{F}}_{{\text{E}}} }} \left( {\text{t}} \right) = \underline {0}$$

Its relevant terms are defined as follows:

8a,b $$\underline{{\theta_{{\text{f}}} }} \left( {\text{t}} \right) = \underline{{\underline {\text{H}}}} \underline{{\theta_{{\text{c}}} }} ,\quad \underline{{\theta_{{\text{f}}} }} \left( {\text{t}} \right) = \left[ {\begin{array}{*{20}c} {{\uptheta }_{{\text{f}}} \left( {t_{0} } \right)} & {{\uptheta }_{{\text{f}}} \left( {t_{1} } \right)} & \cdots & {{\uptheta }_{{\text{f}}} \left( {t_{m - 2} } \right)} & {{\uptheta }_{{\text{f}}} \left( {t_{m - 1} } \right)} \\ \end{array} } \right]^{{\text{T}}},$$

8c $$\underline{{{\uptheta }_{{\text{c}}} }} = \left[ {\begin{array}{*{20}c} {\theta_{m} } & {\theta_{a\left( 1 \right)} } & {\theta_{b\left( 1 \right)} } & \cdots & {\theta_{a\left( k \right)} } & {\theta_{b\left( k \right)} } & \cdots & {\theta_{{a\left( {\eta N_{\max } } \right)}} } & {\theta_{{b\left( {\eta N_{\max } } \right)}} } \\ \end{array} } \right]^{{\text{T}}},$$

8d $$\underline{{\underline {H} }} = \left[ {\begin{array}{*{20}c} 1 & \cdots & {\cos \left( {k\psi_{0} } \right)} & {\sin \left( {k\psi_{0} } \right)} & \cdots \\ 1 & \cdots & {\cos \left( {k\psi_{1} } \right)} & {\sin \left( {k\psi_{1} } \right)} & \cdots \\ {} & \ddots & {} & {} & \ddots \\ 1 & \cdots & {\cos \left( {k\psi_{N - 2} } \right)} & {\sin \left( {k\psi_{N - 2} } \right)} & \cdots \\ 1 & \cdots & {\cos \left( {k\psi_{N - 1} } \right)} & {\sin \left( {k\psi_{N - 1} } \right)} & \cdots \\ \end{array} } \right],\underline{{\underline {H} }}^{\prime } = \omega \underline{\underline{{{\text{HP}}}}} ,\underline {H}^{\prime \prime } = - \omega^{2} \underline{\underline{{{\text{HP}}}}}^{\prime \prime },$$

8e,f $$\underline{{\underline {\text{P}}}}^{{^{\prime}}} = \left[ {\begin{array}{*{20}c} {\begin{array}{*{20}c} 0 & {} \\ {} & \ddots \\ \end{array} } & {\begin{array}{*{20}c} {} & {} \\ {} & {} \\ \end{array} } \\ {\begin{array}{*{20}c} {} & {} \\ {} & {} \\ \end{array} } & {\begin{array}{*{20}c} {\left[ {\begin{array}{*{20}c} 0 & k \\ { - k} & 0 \\ \end{array} } \right]} & {} \\ {} & \ddots \\ \end{array} } \\ \end{array} } \right],\underline{{\underline {\text{P}}}}^{{^{\prime\prime}}} = \left[ {\begin{array}{*{20}c} {\begin{array}{*{20}c} 0 & {} \\ {} & \ddots \\ \end{array} } & {\begin{array}{*{20}c} {} & {} \\ {} & {} \\ \end{array} } \\ {\begin{array}{*{20}c} {} & {} \\ {} & {} \\ \end{array} } & {\begin{array}{*{20}c} {\left[ {\begin{array}{*{20}c} {k^{2} } & 0 \\ 0 & {k^{2} } \\ \end{array} } \right]} & {} \\ {} & \ddots \\ \end{array} } \\ \end{array} } \right]$$

Further, the nonlinear and input functions are defined as follows:

9a,b $$\underline{{{\text{f}}_{{\text{n}}} }} \left( {\underline{{{\uptheta }_{{\text{f}}} }} ,\underline{{{\dot{\theta }}_{{\text{f}}} }} } \right) = \underline{{\underline {\text{H}}}} \underline{{{\text{f}}_{{{\text{nc}}}} }} ,\underline{{{\text{F}}_{{\text{E}}} }} \left( {\text{t}} \right) = \underline{{\underline {\text{H}}}} \underline{{{\text{F}}_{{{\text{Ec}}}} }}$$

9c $$\underline{{{\text{f}}_{{{\text{nc}}}} }} = \left[ {\begin{array}{*{20}c} {f_{m} } & {f_{a\left( 1 \right)} } & {f_{b\left( 1 \right)} } & \cdots & {f_{a\left( k \right)} } & {f_{b\left( k \right)} } & \cdots & {f_{{a\left( {\eta N_{\max } } \right)}} } & {f_{{b\left( {\eta N_{\max } } \right)}} } \\ \end{array} } \right]^{{\text{T}}} ,$$

9d $$\underline{{{\text{F}}_{{{\text{Ec}}}} }} = \left[ {\begin{array}{*{20}c} {F_{m} } & {F_{a\left( 1 \right)} } & {F_{b\left( 1 \right)} } & \cdots & {F_{a\left( k \right)} } & {F_{b\left( k \right)} } & \cdots & {F_{{a\left( {\eta N_{\max } } \right)}} } & {F_{{b\left( {\eta N_{\max } } \right)}} } \\ \end{array} } \right]^{{\text{T}}} .$$

Within these equations, the relevant variables include $\varpi t = \psi$, the nondimensionalized time scale; $\varpi = \omega /\omega_{n}$, the normalized frequency value with a natural frequency of $\omega_{n}$; $T = \eta \tau$, the concerned time period with respect to $0 \le t < T$ → $0 \le \psi < 2\pi /\omega_{n}$; $\eta$, a sub-harmonic index; $\tau$, a fundamental excitation frequency; and *k*, the incremental index defined by $k = \omega_{n} ,{ }2\omega_{n} ,{ }3\omega_{n} \ldots$. By employing the relationship between $\dot{\theta }\left( t \right) = \frac{d\theta }{{dt}} = \varpi \frac{d\theta }{{d\psi }} = \varpi \theta^{\prime}$ and $\ddot{\theta }\left( t \right) = \varpi^{2} \theta^{\prime\prime}$, the overall Galerkin scheme of Eq. (7) is expressed as follows:

10a,b $$\underline{\underline{H}} \underline {\Psi } = \underline {0} ,\underline {\Psi } = - \varpi^{2} m\underline{\underline{{\text{P}}}}^{\prime \prime } \underline{{\theta_{{\text{c}}} }} + \varpi c\underline{\underline{{\text{P}}}}^{\prime } \underline{{\theta_{{\text{c}}} }} + \underline{{{\text{f}}_{{{\text{nc}}}} }} - \underline{{{\text{F}}_{{{\text{Ec}}}} }} = \underline {0}$$

To determine the solutions of $\underline{{{\uptheta }_{{\text{c}}} }}$ and $\varpi$ for each step, the Newton–Raphson method was implemented under the condition $\underline {{\Psi }} \to \underline {0}$, where $\underline {{\Psi }}$ is considered as a function of $\underline{{{\uptheta }_{{\text{c}}} }}$ and $\varpi$ such that $\underline {{\Psi }}\left( {\underline{{{\uptheta }_{{\text{c}}} }} ,\varpi } \right)$. Prior studies can be referred to for more derivation and descriptions on the HBM^8^.

### Investigation of sub-harmonic responses under the frequency up-sweeping condition

**Figure 3:**
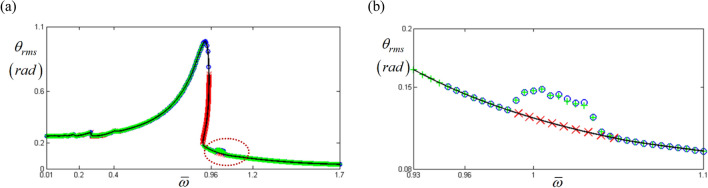
Nonlinear frequency response with RMS under frequency up-sweeping stability conditions: (**a**) comparison of the HBM with NS; (**b**) comparison of the HBM and NS at sub-harmonic resonance. Key: Blue Open circle, NS with frequency up-sweeping; Green plus, NS with frequency down-sweeping; Black line, HBM result; Red times, HBM result under the unstable condition^8^.

Figure 3 shows a comparison of the HBM and NS which was also studied in the prior study^8^. The HBM was conducted with $\eta$ = 2 and *N*_*max*_ = 12 under the frequency up-sweeping condition, Hill’s method was utilized to determine the stability condition^4,8,17,24^, and the valid components of the input torque vector were expressed as $F_{m} = 168.9$, $F_{a\left( 2 \right)} = - 87.97$, and $F_{b\left( 2 \right)} = 235.65$ in Eq. (9d)^8,22,23^. Figure 3 also presents a correlation between the simulations resulting from the HBM and NS; however, the outcome of both at the sub-harmonic region marked with a red dotted line, as shown in Fig. 3b, are not matched with each other; the NS results simulate the sub-harmonic resonance well, while the HBM follows the normal harmonic response path with an indication of instability.

**Figure 4:**
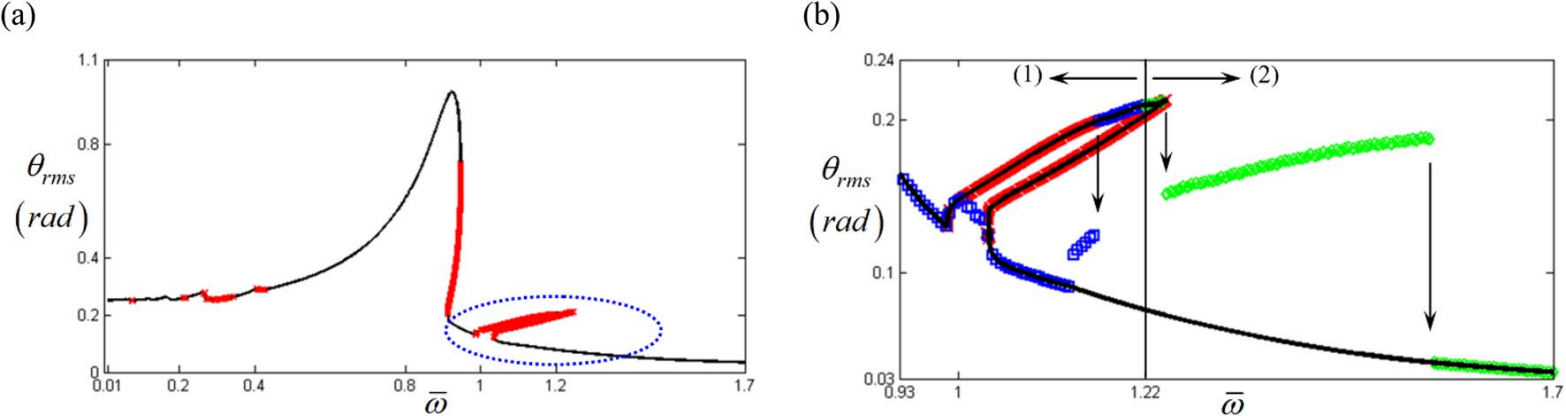
Nonlinear frequency response with RMS reflecting sub-harmonic resonance: (**a**) the HBM result under unstable conditions with sub-harmonic resonance; (**b**) comparison of HBM with NS by focusing on the specific stable regime. Key: Black line, HBM result; Red times**,** HBM result under unstable conditions; Blue square, NS results with frequency down-sweeping; Green diamond, NS results with frequency up-sweeping.

Based on a previous study^18^, numerical techniques must exist to determine sub-harmonic resonant behaviors; hence, to reveal these sub-harmonic responses, the input torque values pertaining to the sub-harmonic locations, such as $F_{a\left( 1 \right)}$ and, $F_{b\left( 1 \right)}$ were given by $F_{a\left( 1 \right)} = \varepsilon F_{a\left( 2 \right)}$ and $F_{b\left( 1 \right)} = \varepsilon F_{b\left( 2 \right)}$. Here, $1 \times 10^{ - 5}$ is artificially employed for $\varepsilon$ to avoid triggering the system responses corresponding to the normal input torque. Figure 4a shows that the simulated results successfully included sub-harmonic resonances marked with blue dotted circles. In general, the unstable conditions of the HBM are closely related to complex dynamic behaviors, such as super- or sub-harmonic resonances and bifurcations. However, the stable conditions always indicate responses from the practical system that follow their relevant excitation harmonics such as in Fig. 4b, where a specific region under the stable condition is observed around the concentrated sub-harmonic resonance area at $\varpi = 1.22$. To examine its nonlinear dynamic behaviors, a NS was conducted by artificially applying the initial conditions, $\theta_{f} \left( 0 \right) = - 1.89{ }\left( {rad} \right)$ and $\dot{\theta }_{f} \left( 0 \right) = - 1.04{ }\left( {rad/s} \right)$, with the assumption that these conditions can be abruptly changed physically. The blue rectangles and the green diamonds indicated in Fig. 4b show the frequency down- and up-sweeping results, respectively, from the stable region in the sub-harmonic resonance at $\varpi = 1.22$. Furthermore, the areas marked with (1) and (2) in Fig. 4b show the additional system results from the NS when compared with the HBM, indicating that the nonlinear responses in this regime are affected by more sub-harmonic resonances. In addition, both results based on the HBM and NS could not show all of the possible solutions that appear in Fig. 4b since the solutions are much dependent on the initial conditions which reflect the solutions calculated at the prior steps.

**Figure 5:**
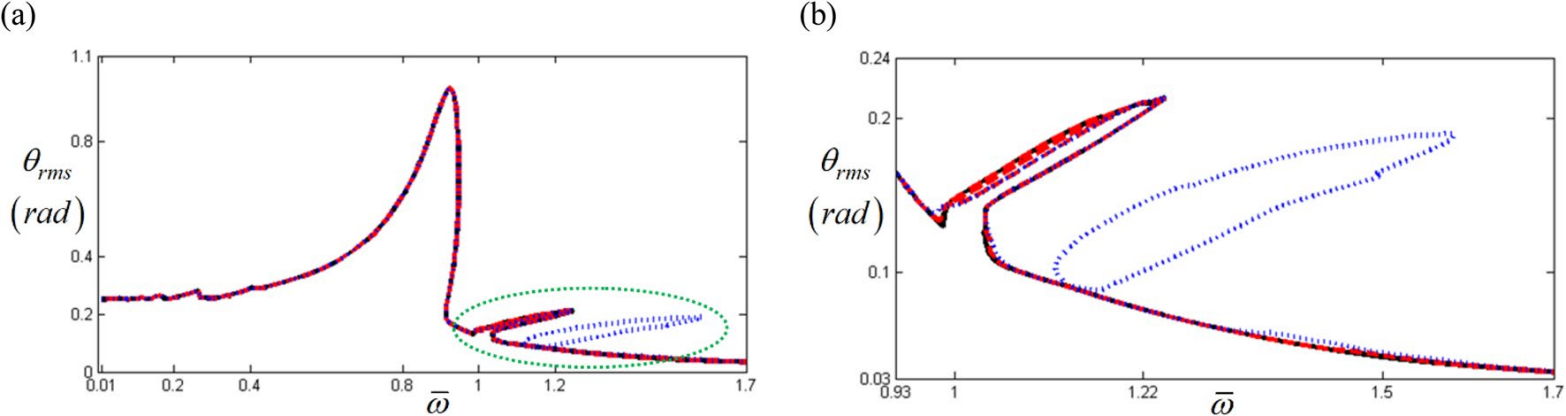
Comparison of HBM results under various excitation conditions: (**a**) the HBM results under frequency up-sweeping; (**b**) comparison of sub-harmonic responses under various excitations. Key: Black line, HBM result with $\varepsilon = 1 \times 10^{ - 5}$; Red dashed line HBM result with $\varepsilon = 1 \times 10^{ - 3}$; Blue dotted line, HBM result with $\varepsilon = 2.9 \times 10^{ - 3}$.

**Figure 6:**
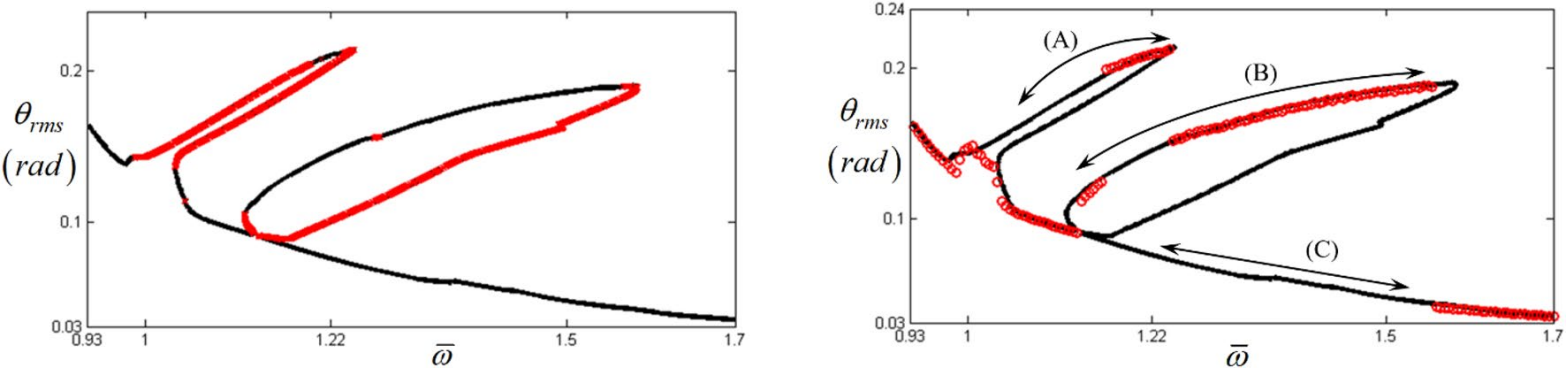
Nonlinear frequency response with RMS at sub-harmonic resonances: (**a**) HBM results with stability conditions; (**b**) comparison of HBM and NS results with various initial condition paths. Key: Balck line, HBM result with $\varepsilon = 2.9 \times 10^{ - 3}$; Red times, HBM results with the unstable condition; Red open circle, NS result.

To capture all the relevant sub-harmonic resonances in this regime, the appropriate input torque values for the sub-harmonic components in Eq. (9c) can be artificially obtained using various values of $\varepsilon$. To trigger their sub-harmonic resonances, $\varepsilon$ must be increased within a certain range of values assumed to be nearly zero numerically. Figure 5 compares the three results of the HBM using $1 \times 10^{ - 5}$, $1 \times 10^{ - 3}$ and $2.9 \times 10^{ - 3}$ as values of $\varepsilon$. Assuming the baseline is the HBM simulation with $\varepsilon = 1 \times 10^{ - 5}$, the results with $\varepsilon = 1 \times 10^{ - 3}$ and $\varepsilon = 2.9 \times 10^{ - 3}$ follow the nonlinear system response of the baseline well. However, as displayed in Fig. 5, the simulation with $\varepsilon = 2.9 \times 10^{ - 3}$ was the only one to successfully capture all sub-harmonic resonances. Figure 6a shows the stability conditions with the arc-length continuation schemes, and Fig. 6b compares the two results from the HBM and NS. The latter are calculated using the initial conditions at $\varpi = 1.22$ of the upper sub-harmonic resonance regime and three different solution paths are presented, with paths (A), (B), and (C) defined in this study as the upper, lower, and normal paths. Complex dynamic behaviors in sub-harmonic resonant areas can be analyzed using information shown in Fig. 6b. First, as indicated in Fig. 3, the NS with frequency up-sweeping from $\varpi = 0.93$ and frequency down-sweeping from $\varpi = 1.7$, follows path (C). Second, the nonlinear solutions on paths (A) and (B) can be obtained by utilizing each stable regime’s particular initial conditions, as shown in Fig. 6a. Third, the dynamic behaviors between $\varpi = 1$ and $\varpi = 1.7$ are sensitive to various initial conditions; hence, the system responses can be abruptly changed among paths (A), (B), and (C). The initial conditions of a physical system can be determined depending on various external factors, such as sudden changes in drag torques and velocities due to fluctuations in road conditions. For example, vibro-impacts such as gear rattles occur with different driving conditions^23^. While gear rattles generally arise around acceleration ranges of 1800 rpm, the same vibro-impacts are also found at higher velocity ranges of 3200 rpm when the system is under coast conditions. This, as shown in the nonlinear resonant issues in Fig. 6, demonstrates that the same dynamic behaviors can occur under different initial conditions.

**Figure 7:**
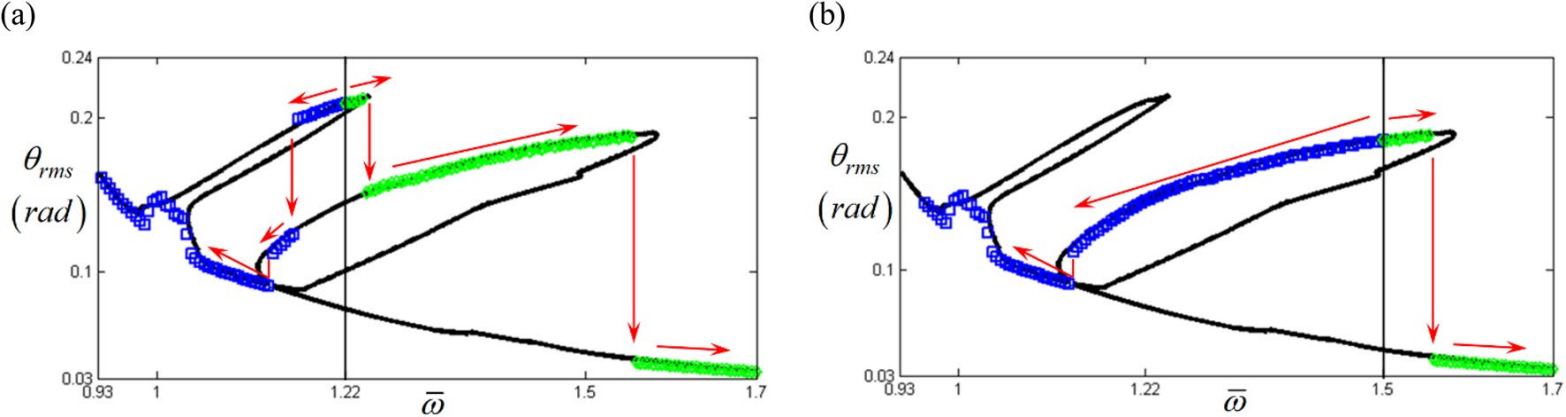
Comparison of HBM with NS under various initial conditions: (**a**) comparison of HBM with NS calculated at $\varpi = 1.22$; (**b**) comparison of HBM with NS calculated at $\varpi = 1.5$. Key: Black line, HBM result with $\varepsilon = 2.9 \times 10^{ - 3}$; Blue square, NS results with frequency down-sweeping; Green diamond, NS results with frequency up-sweeping.

Figure 7a and b compare two results under two different initial conditions. A comparison of the calculated HBM and NS reveals that the solutions for path (A) under the initial conditions at $\varpi = 1.22$ follow all paths (A), (B), and (C), and are designated as stable solutions of the HBM. When the initial conditions for path (B) have $\theta_{f} \left( 0 \right) = - 0.15{ }\left( {rad} \right)$ and $\dot{\theta }_{f} \left( 0 \right) = - 12.54{ }\left( {rad/s} \right)$ at $\varpi = 1.5$, the NS results only follow paths (B) and (C), and never appear on path (A). These dynamic characteristics are clearly shown in Figs. 6 and 7.

### Examination of bifurcation characteristics with the real part of eigenvalues

As shown in Figs. 3, 4, and 6, the stability conditions, which were determined during the HBM, are examined, and the real parts of the eigenvalues are considered. Applying the basic procedures of Hill’s method, the system responds as follows.

11a,b $$\underline{{{\uptheta }_{{\text{f}}} }} \left( {\text{t}} \right) = \underline{{{\uptheta }_{{{\text{fp}}}} }} \left( {\text{t}} \right) + \underline{{{\upxi }_{{\text{f}}} }} \left( {\text{t}} \right),\quad \underline{{{\upxi }_{{\text{f}}} }} \left( {\text{t}} \right) = \underline {\text{p}}\left( {\text{t}} \right)e^{\lambda t}$$

**Figure 8:**
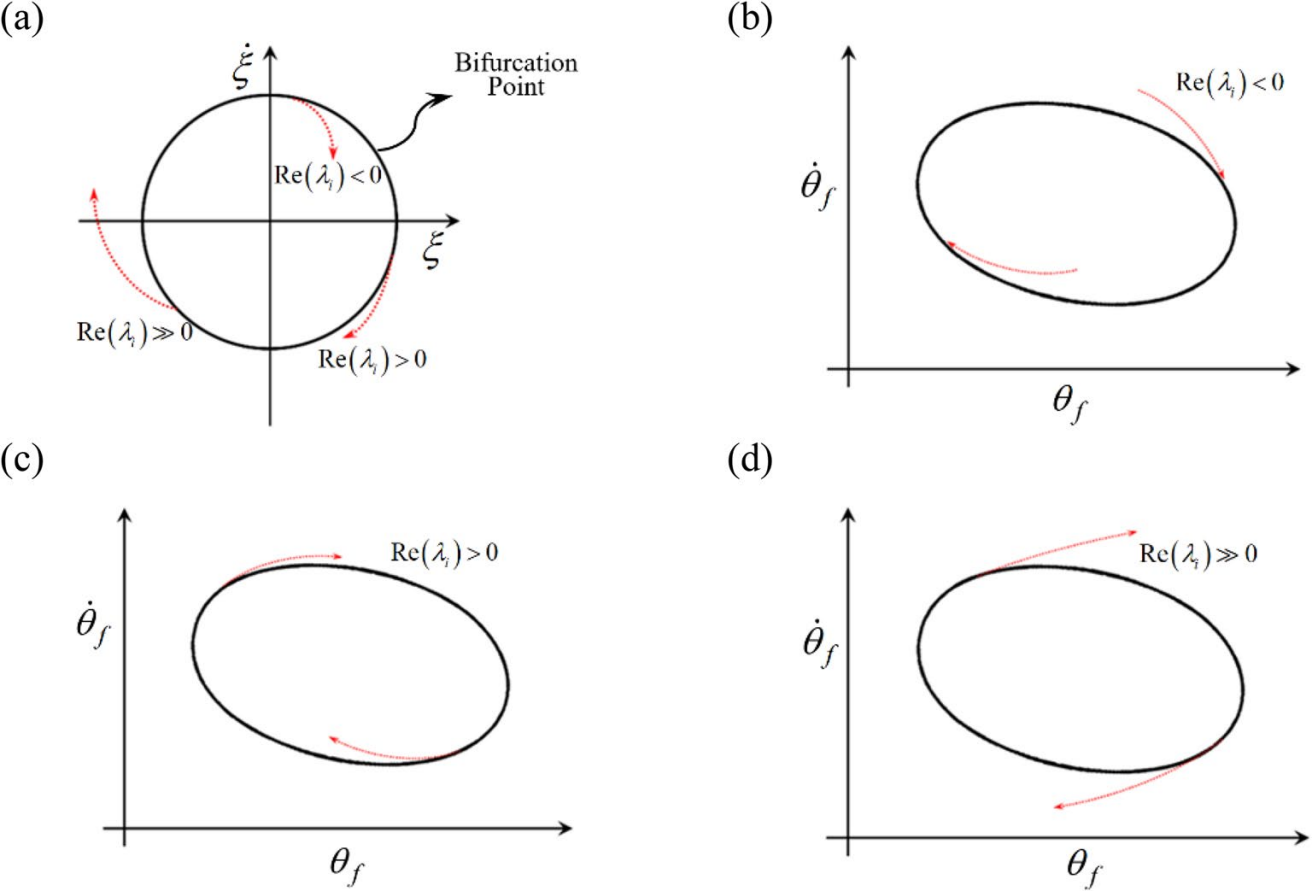
Nonlinear dynamic behaviors along with the various real parts of eigenvalues: (**a**) bifurcation points and various branches of transient responses; (**b**) system response under ${\text{Re}} \left( {\lambda_{i} } \right) < 0$; (**c**) system response under ${\text{Re}} \left( {\lambda_{i} } \right) > 0$; (**d**) system response under ${\text{Re}} \left( {\lambda_{i} } \right) \gg 0$.

Here, $\underline{{{\uptheta }_{{\text{f}}} }} \left( {\text{t}} \right)$ and $\underline{{{\upxi }_{{\text{f}}} }} \left( {\text{t}} \right)$ are the periodic (particular) and perturbation (homogeneous) parts of the solutions, respectively^4,8,22^, and $\underline{{{\upxi }_{{\text{f}}} }} \left( {\text{t}} \right)$ consists of $\underline {\text{p}}\left( {\text{t}} \right)$ and $e^{\lambda t}$, the periodic and decay terms, respectively. Based on the calculated eigenvalues $\lambda_{i}$ with $i = 1,{ }2, \ldots ,{ }2\left( {2\eta N_{\max } + 1} \right)$, at least one positive value among ${\text{Re}} \left( {\lambda_{i} } \right)$ makes the system unstable. In addition,${\text{Re}} \left( {\lambda_{i} } \right) = 0$ satisfies the condition that the system responses fall into bifurcation, as indicated in Fig. 8a^7,24^. From this point onward, this study assumes that the properties of the positive ${\text{Re}} \left( {\lambda_{i} } \right)$ signify the bifurcation characteristics, and by focusing on the frequency up-sweeping direction, this bifurcation is assumed to occur in the regimes determined as unstable. Thus, only these unstable regimes will be examined, with the exception of the arc-length solutions’ reverse direction. Though various conditions of ${\text{Re}} \left( {\lambda_{i} } \right)$ are investigated, they are generally considered in three forms: ${\text{Re}} \left( {\lambda_{i} } \right) < 0$, ${\text{Re}} \left( {\lambda_{i} } \right) > 0$, and ${\text{Re}} \left( {\lambda_{i} } \right) \gg 0$. Under the condition ${\text{Re}} \left( {\lambda_{i} } \right) < 0$, its relevant transient responses $\underline{{{\upxi }_{{\text{f}}} }} \left( {\text{t}} \right)$ reach zero, which stabilizes the system response and sustains the particular solution, $\underline{{{\uptheta }_{{\text{f}}} }} \left( {\text{t}} \right) = \underline{{{\uptheta }_{{{\text{fp}}}} }} \left( {\text{t}} \right)$ as depicted in Fig. 8b. However, ${\text{Re}} \left( {\lambda_{i} } \right) > 0$ and ${\text{Re}} \left( {\lambda_{i} } \right) \gg 0$ cause the system to fall into diverging conditions by preventing the system responses from maintaining that particular solution, as illustrated in Fig. 8c and d. ${\text{Re}} \left( {\lambda_{i} } \right) > 0$, rather than ${\text{Re}} \left( {\lambda_{i} } \right) \gg 0$, is assumed to gradually diverge the system response from the periodic solution of $\underline{{{\uptheta }_{{\text{f}}} }} \left( {\text{t}} \right) = \underline{{{\uptheta }_{{{\text{fp}}}} }} \left( {\text{t}} \right)$; hence, it is assumed that ${\text{Re}} \left( {\lambda_{i} } \right) > 0$ is relevant for period-doubling or period-halving cascades. On the other hand, it is assumed that ${\text{Re}} \left( {\lambda_{i} } \right) \gg 0$ allows the system responses to change rapidly into more complicated phenomena, such as quasi-periodic and chaotic.

**Figure 9:**
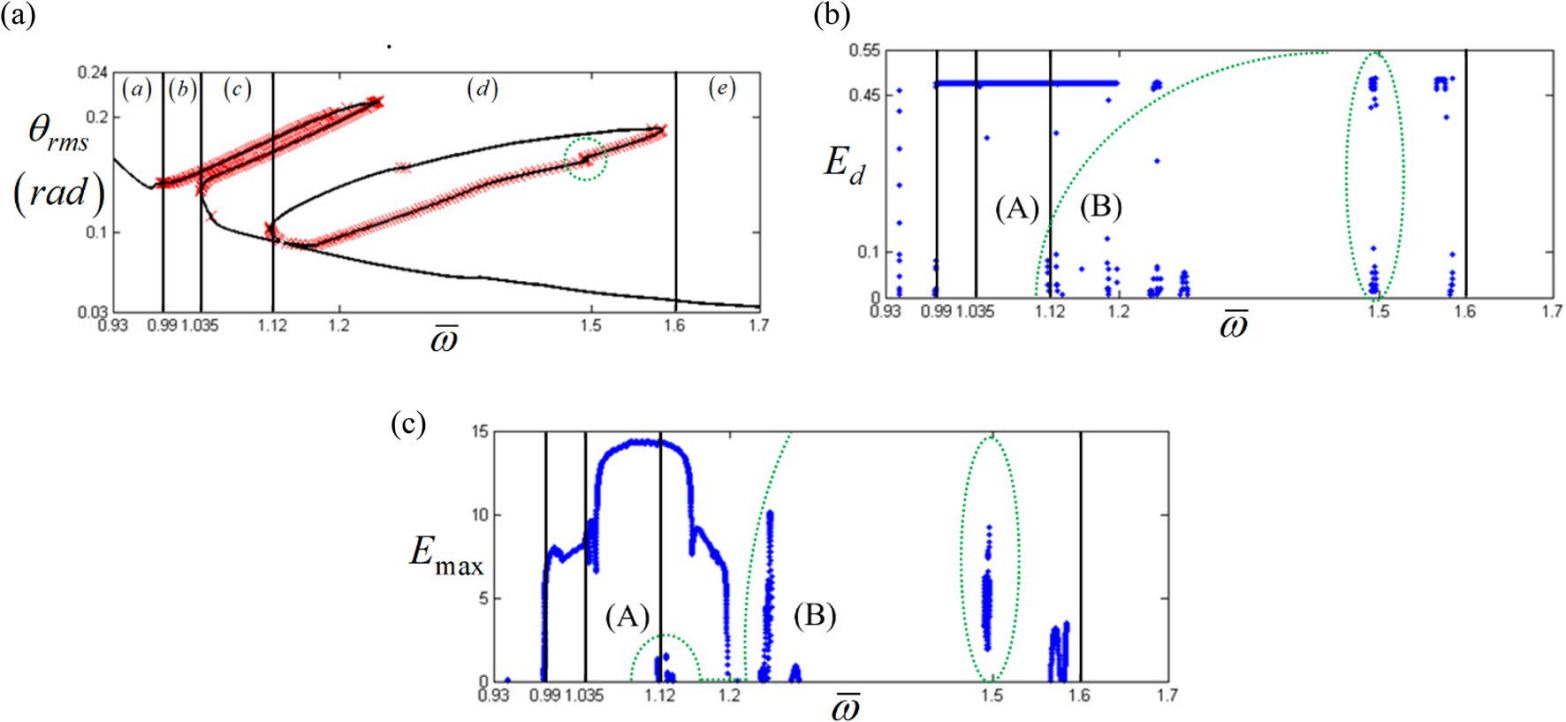
HBM result under unstable conditions compared with the properties of ${\text{Re}} \left( {\lambda_{i} } \right)$: (**a**) HBM result at sub-harmonic resonances with various specific areas; (**b**) distribution of positive ${\text{Re}} \left( {\lambda_{i} } \right)$ along with different solution paths; (**c**) maximum value of positive ${\text{Re}} \left( {\lambda_{i} } \right)$ along with different solution paths. Key: Black line, HBM result; Red times**,** HBM result with unstable condition.

The relationships between each unstable solution of the HBM and the properties of ${\text{Re}} \left( {\lambda_{i} } \right)$ are shown in Fig. 9. To implement the previously suggested ideas, two properties, *E*_*d*_ the distribution of positive ${\text{Re}} \left( {\lambda_{i} } \right)$ and *E*_*max*_ the maximum value of positive ${\text{Re}} \left( {\lambda_{i} } \right)$ can be considered. Here, *E*_*d*_, from whole number values of ${\text{Re}} \left( {\lambda_{i} } \right)$, is defined as follows:

12 $$E_{d} = \frac{{Number \,of\, positive\, {\text{Re}} \left( {\lambda_{i} } \right)}}{{Overall \,number\, of\, {\text{Re}} \left( {\lambda_{j} } \right)}}\,with\,i, j = 1, 2, \ldots$$

**Figure 10:**
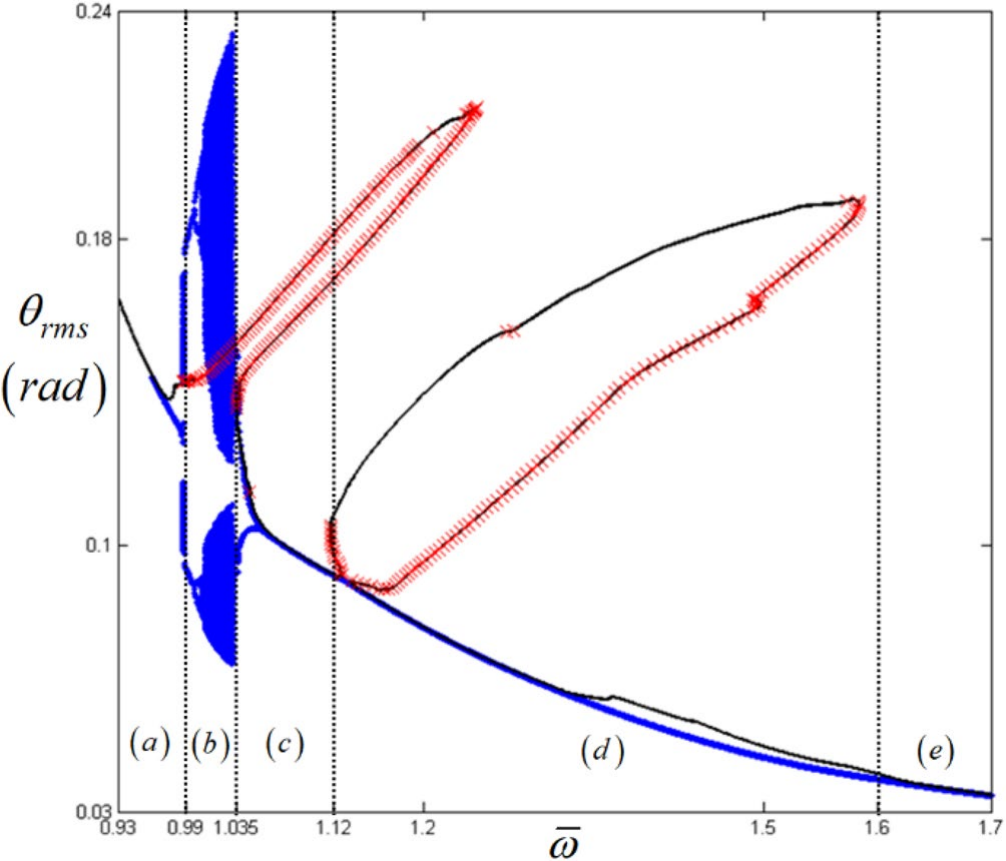
Comparison of HBM and bifurcation diagram with RMS values focused on super-harmonic resonances with the initial condition path (C): Key: Black line, HBM result with stable condition; Red times**,** HBM result with unstable condition; Blue circle, bifurcation diagrams.

**Figure 11:**
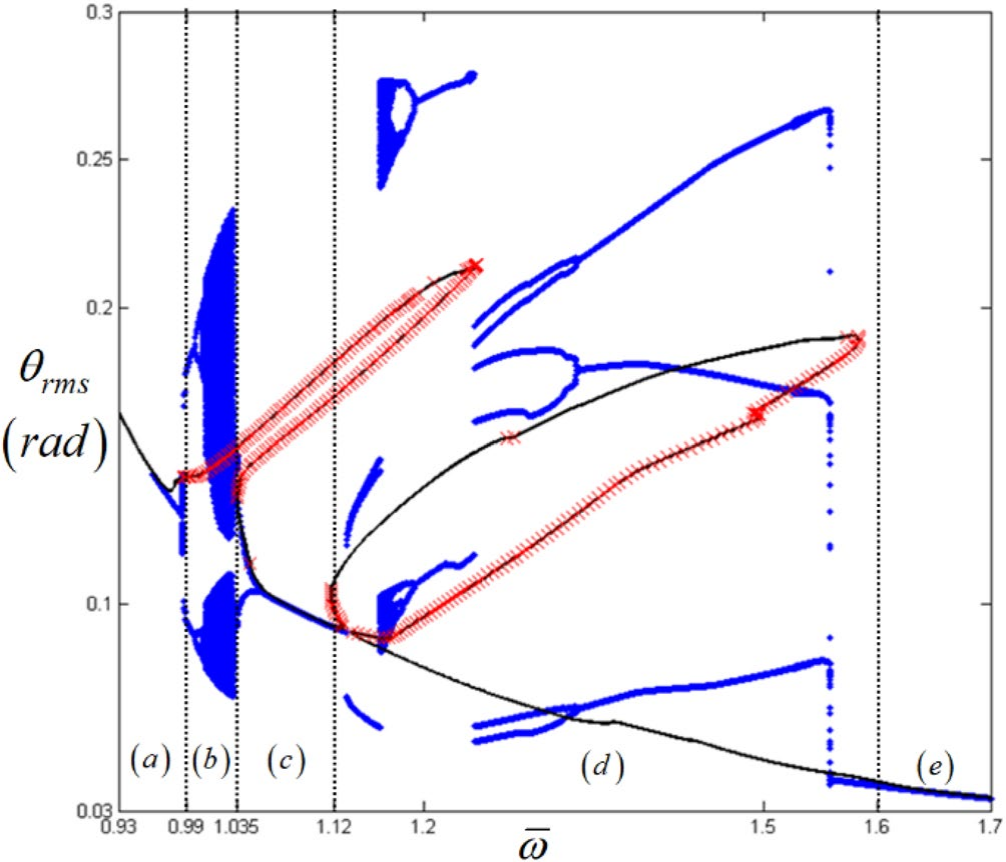
Comparison of HBM and bifurcation diagram with RMS values focused on sub-harmonic resonances with the initial condition path (A): Key: Black line, HBM result with stable condition; Red times**,** HBM result with unstable condition; Blue circle, bifurcation diagrams.

**Figure 12:**
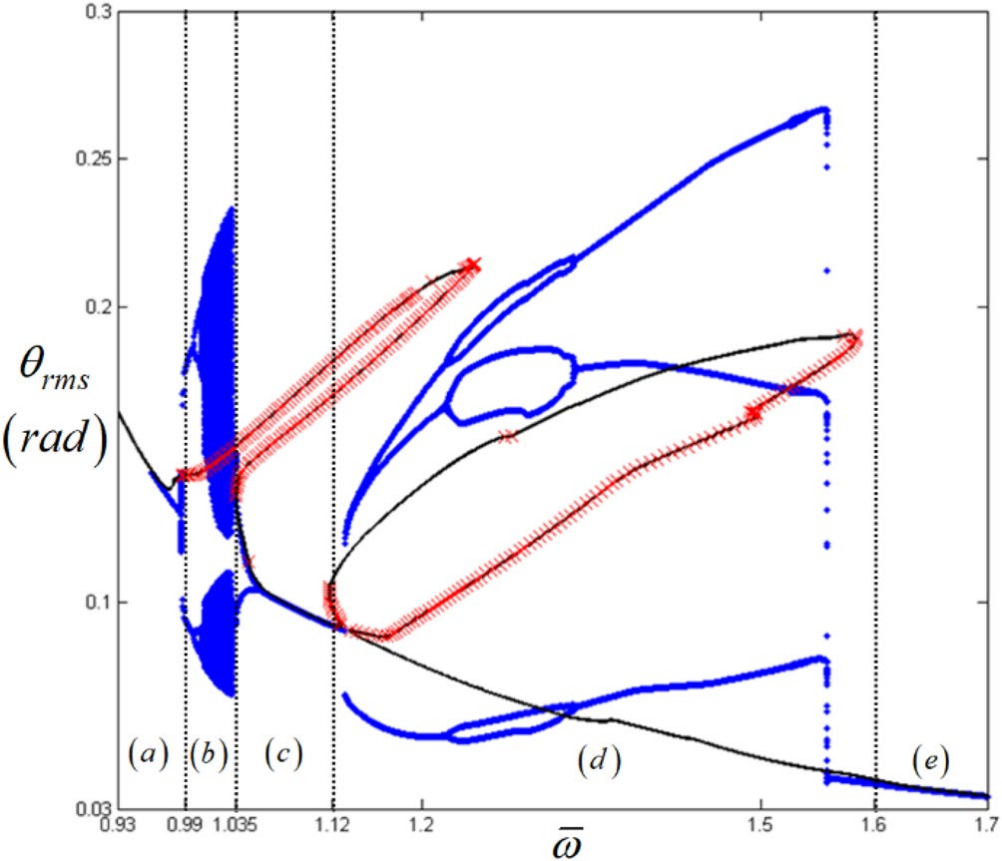
Comparison of HBM and bifurcation diagram with RMS values focused on sub-harmonic resonances with the initial condition path (B): Key: Black line, HBM result with stable condition; Red times**,** HBM result with unstable condition; Blue circle, bifurcation diagrams.

Figure 9 also illustrates regimes (a)–(e), which has been carefully examined as follows. Regimes (a) and (e) are the stable response areas where no *E*_*d*_ and *E*_*max*_ are obtained, and the small numbers of *E*_*d*_ in regime (a) are related to the unstable conditions of the primary resonance, not the sub-harmonic resonance. When regime (b) is examined, the value of *E*_*d*_ is higher than 0.45 and the value of *E*_*max*_ is higher than 5, and the system responses are much more complex with quasi-periodic or chaotic motions. Moreover, the system responses are smoothly connected between regimes (a) and (c), even though it follows the arc-length path (A), as indicated in Fig. 6b. Since the two stable regions in (a) and (c) are located near the unstable responses of (b), instead of obeying the arc-length path (A), the dynamic behaviors occur by following the order of: (1) a stable response in regime (a), (2) an unstable response in regime (b), and (3) a stable response in regime (c). Due to a series of smooth changes in the stability conditions from regimes (a) to (c), regime (c) is expected to be under pure harmonic responses, and thus, the calculated results of *E*_*d*_ and *E*_*max*_ can only be ignored for regime (c). Additionally, the characteristics of the bifurcation diagrams observed in Figs. 10, 11, and 12, also correlate well when focused on regimes (a)–(c) in particular. The green dotted circles and ellipses in regime (d) are the reverse path results of those for the lower branch of sub-harmonic resonance, which is not the main concern of this study.

An examination of the calculated values *E*_*d*_ and *E*_*max*_ must be carefully carried out when investigating the nonlinear dynamic behaviors for regime (d), since these values are taken from two different paths (A) and (B), as shown in Fig. 6b. When the bifurcation characteristics shown in Figs. 11 and 12 are compared, clear differences in the two system responses are observed depending on the initial conditions. For example, Fig. 11 shows the results when the initial conditions are set at ${ }\varpi = 1.22$ on path (A), as shown in Fig. 4. From this point, the solutions follow the frequency down-sweeping direction, and first show the period-halving and period-halving cascades which fall into chaotic in regime (d). When the solutions follow the frequency up-sweeping direction, they enter the period-doubling cascade and finally reach the pure harmonic response regime. When these complex bifurcation phenomena are compared with Fig. 9b and c, each bifurcation characteristic is correlated with the calculated values of *E*_*d*_ and *E*_*max*_, marked by green dotted lines, based on their relevant sub-harmonic resonances, as indicated by (A) and (B). On the other hand, compared with Figs. 11, 12 presents simpler responses, as also seen in Fig. 7b; thus, the bifurcations only occur for the arc-length paths (B) and (C). The two bifurcation diagrams in Figs. 11 and 12, with the exception of the responses around the area at $\varpi = 1.22{ }$ are correlated with each other and through careful examination of Fig. 9, 10, 11 and 12 the results of regimes (c) and (d) provide the basic ideas required to map the complex nonlinear dynamic behaviors. First, the system responses can be abruptly changed under various initial conditions, and should follow different solution paths, despite the potential for a smooth change in the initial conditions along the normal solutions path (C), as shown in Fig. 6. Second, the bifurcation characteristics can also be defined under various initial conditions and can be anticipated along with the relevant eigenvalues *E*_*d*_ and *E*_*max*_ in unstable conditions, as shown in Fig. 9.

## Conclusion

This study investigated the nonlinear dynamic behaviors in sub-harmonic resonant areas under various initial conditions. Depending on these different initial conditions, the system responses followed different paths of the arc-length solutions from the HBM. Regarding the complex nonlinear phenomena, the contributions of this research are as follows. First, this study suggested a method to determine all possible sub-harmonic resonances by employing various excitation conditions, successfully simulating two branches of sub-harmonic resonances using the HBM with a variety of initial conditions. Second, this study investigated various paths of nonlinear responses under different initial conditions by employing the HBM in comparison with the NS. These results show the multitude of system responses, while the dynamic behaviors follow different arc-length solution paths. Finally, the bifurcation characteristics that occur in the unstable solutions were examined based on the eigenvalues in terms of *E*_*d*_ and *E*_*max*_. This provided basic ideas for predicting the bifurcation characteristics numerically.

The current study has dealt with a simple torsional system with 1DOF which is a part of whole driveline system^23^. As a further study, the conceptual approach employed for this study can be extended to investigate the severe vibro-impact problems which occur in the driveline system concerned with the gear rattle or the gear whine.

## Data Availability

The datasets generated and/or analysed during the current study are not publicly available due to its relatively large size but are available from the corresponding author on reasonable request.
